# Genomic-based transmission analysis of carbapenem-resistant *Pseudomonas aeruginosa* at a tertiary care centre in Cologne (Germany) from 2015 to 2020

**DOI:** 10.1093/jacamr/dlac057

**Published:** 2022-05-20

**Authors:** Andreas F. Wendel, Monika Malecki, Frauke Mattner, Kyriaki Xanthopoulou, Julia Wille, Harald Seifert, Paul G. Higgins

**Affiliations:** 1 Institute of Hygiene, Cologne Merheim Medical Centre, University Hospital of Witten/Herdecke, Cologne, Germany; 2 Division of Hygiene and Environmental Medicine, Department of Human Medicine, Faculty of Health, Witten/Herdecke University, Witten, Germany; 3 Institute for Medical Microbiology, Immunology and Hygiene, Faculty of Medicine and University Hospital Cologne, University of Cologne, Cologne, Germany; 4 German Centre for Infection Research, Partner site Bonn-Cologne, Cologne, Germany

## Abstract

**Objectives:**

To describe the propensity of carbapenem-resistant *Pseudomonas aeruginosa* to spread within a hospital critical care setting.

**Methods:**

The study was conducted in a 700-bed tertiary centre in Cologne, Germany. *P. aeruginosa* resistant to piperacillin, ceftazidime, cefepime, imipenem, meropenem and ciprofloxacin, isolated from clinical and screening specimens from four critical care units from 2015 to 2020 were analysed. Genotyping was carried out by WGS (Illumina and MinION). MLST, core genome MLST (cgMLST) and resistome analysis was performed and merged with epidemiological data.

**Results:**

Fifty-five out of 79 non-duplicate *P. aeruginosa* isolates were available, of which 20 were carbapenemase producers as follows: *bla*_VIM-1_ (*n *= 1), *bla*_VIM-2_ (*n *= 17), *bla*_VIM-4_ (*n *= 1), and *bla*_NDM-1_/*bla*_GES-5_ (*n *= 1). Forty-two of 55 isolates were hospital-acquired. cgMLST revealed three clusters: Cluster 1 (*n *= 15, ST111, *bla*_VIM-2_, recovered between 2015 and 2020); Cluster 2 (*n *= 4, ST970, carbapenemase negative); and Cluster 3 (*n *= 2, ST357, carbapenemase negative). The *bla*_VIM-2_ gene of Cluster 1 was integrated on the chromosome in a class 1 integron (type In59). Using conventional epidemiology, we were only able to confirm two patient-to-patient transmissions and one room-to-patient transmission on three different ICUs within Cluster 1. Isolates from Cluster 2 represented an outbreak occurring in 2019.

**Conclusions:**

These data give insight into the epidemiology of carbapenem-resistant *P. aeruginosa*. Transmission dynamics differed between carbapenemase- and non-carbapenemase-producing isolates. A continuous acquisition of clonally related ST111 VIM-2 *P. aeruginosa*, being the main carbapenemase-producing strain, was observed over the whole study period, as well as an overall higher genomic diversity among non-carbapenemase-producing *P. aeruginosa*.

## Introduction

*Pseudomonas aeruginosa* is an environmental bacterium that can colonize the human body. As a leading nosocomial pathogen *P. aeruginosa* may cause surgical site infections, ventilator-associated pneumonia, catheter-associated urinary tract infections or central-line-associated bloodstream infections in healthcare settings.^1^ The organism is especially problematic for immunocompromised patients within special units (ICU, haematology-oncology ward or burn unit).^2^ Infections can be difficult to treat because of intrinsic resistance to many antimicrobial agents as well as rapid development of antimicrobial resistance to nearly all available antimicrobials through chromosomal mutations and acquisition of transferable resistance genes.^3^ Of particular interest is carbapenem resistance mediated by intrinsic resistance mechanisms (a combination of efflux pumps, AmpC overexpression and porin loss) or acquisition of a carbapenemase, especially an MBL.^3^ Carbapenemase production is linked to globally distributed and emerging MDR or even XDR high-risk clones.^4,5^ While the emergence of carbapenem-resistant *P. aeruginosa* is well described, less is known about the different propensity of carbapenemase-producing and carbapenemase-non-producing *P. aeruginosa* to spread within the hospital setting. This is important, as a relevant part of hospital-acquired infections caused by *P. aeruginosa* is transmission-associated, either patient-to-patient (mostly via the hands of healthcare workers) or environment-to-patient.^6,7^ The hospital environmental is a known reservoir, especially in moist sites. Reports show that contaminated tap water as well as washbasins are linked to transmission events.^8–11^

A previous study from our research group performed in three hospitals of different levels of healthcare has shown a prevalence of approximately 20% carbapenemase producers, mostly VIM-2, amongst MDR/XDR *P. aeruginosa* over a 3 year period.^12^ Using PFGE nearly all VIM-2-producing isolates were clonally related. However, only a few epidemiologically proven transmission events were confirmed, exclusively on several ICUs of the tertiary care centre.^12^ The present investigation aims to define the local epidemiology and transmission dynamics of MDR/XDR *P. aeruginosa* irrespective of carbapenemase production in these ICUs of the tertiary care centre over a period of 5 years using a genomics-based approach.

## Materials and methods

### Study setting

The study was conducted in a 700-bed tertiary care centre in Cologne, Germany. Based on data of the implemented active surveillance system following the protocol of the German healthcare-associated infection surveillance on intensive care units (ITS-KISS)^13^ and previous studies,^12^ four out of six available critical care units with a frequent detection and/or possible transmission events of carbapenem-resistant and MDR/XDR *P. aeruginosa* were chosen: three ICUs (ICU 1–3) and one intermediate care unit (ImCU1). ICU1 and ICU3 provide care for surgical patients including burn patients (max. of 32 and 14 beds, respectively), whereas ICU2 and ImCU1 are primarily reserved for medical patients (max. of 18 and 16 beds, respectively). Overall, there were approximately 3500 admissions (20 000 patient days) per year on these units. The number of patients colonized/infected with *P. aeruginosa* was assessed using the laboratory surveillance information system (Hybase v.6, epiNET AG, Germany).

### Identification and susceptibility testing

All isolates were identified with standard microbiological procedures using the Vitek 2 system (Vitek GN-ID, bioMérieux, Marcy l’Étoile, France) or MALDI-TOF (Bruker Daltonics, Bremen, Germany). First susceptibility testing was performed with automated systems (Vitek 2 system from bioMérieux or the BD-Phoenix system from BD Diagnostics, Heidelberg, Germany) or disc diffusion (BD Sensi-Disc, BD Diagnostics) and later confirmed by broth microdilution using Micronaut-S Pseudomonas MIC panels (Merlin Diagnostika, Bornheim, Germany) according to the manufacturers’ instructions. MICs were determined for piperacillin, piperacillin/tazobactam, ceftazidime, cefepime, ceftazidime/avibactam, ceftolozane/tazobactam, imipenem, meropenem, aztreonam, gentamicin, tobramycin, amikacin, ciprofloxacin, levofloxacin, fosfomycin and colistin. EUCAST breakpoints (v11.0, 2021) were used for interpretation. *P. aeruginosa* resistant to piperacillin, ceftazidime, cefepime, imipenem, meropenem and ciprofloxacin isolated on the designated wards from clinical and screening specimens from January 2015 to June 2020 were included. This basically corresponds to an MDR/XDR phenotype according to the ECDC/CDC classification.^5^ Two VIM-2-producing *P. aeruginosa* isolates detected on other wards of the same hospital analysed in a previous study (PSA-2016-03 and PSA-2017-02) were also included in this study.^12^

### WGS

To prepare short-read sequencing libraries, fresh cultures were grown overnight on Mueller–Hinton agar and DNA was isolated using the DNeasy Ultra Clean Microbial Kit (Qiagen, Hilden, Germany) following the manufacturer’s instructions. Sequencing libraries were prepared with the purified DNA using the Ultra II FS DNA Library Prep Kit (New England Biolabs, Frankfurt, Germany) for a 250 bp paired-end sequencing run on an Illumina MiSeq sequencer. *De novo* assembly was performed using Velvet (version 1.1.04).^14^ The raw sequencing short reads generated in this project were submitted to the European Nucleotide Archive (https://www.ebi.ac.uk/ena/) under the Project Accession number PRJEB43695.

To understand the genetic location of the *bla*_VIM-2_ gene, three strains belonging to the same core genome MLST (cgMLST) cluster (PSA-2015-07, PSA-2017-03 and PSA-2020-04) were selected for long-read sequencing. DNA was extracted from bacteria grown overnight in Luria broth using the Genomic-Tips 100/G kit and Genomic DNA Buffers kit (Qiagen). Libraries were prepared using the Ligation Sequencing Kit (SQK-LSK109) combined with Native Barcoding Kit (EXP-NBD114) and were loaded onto a R9.4 flow cell (Oxford Nanopore Technologies, Oxford, UK) for a MinION sequencing run. Finally, a hybrid assembly of the long- and short-reads was performed using Unicycler.^15^ The long-read raw data have been deposited to the Sequence Read Archive (https://www.ncbi.nlm.nih.gov/sra) under the BioProject Accession number PRJNA771632.

### Genotyping and resistome analysis

Relatedness of all isolates was assessed by a cgMLST genotyping approach. Assembled genomes were analysed by SeqSphere+ software (v.7.2.3, Ridom, Germany) using a validated cgMLST scheme recently proposed by Tönnies *et al.*^16^ and based on 3867 target genes. During comparison of the allelic profile the ‘pairwise ignoring missing values’ option was turned on. Genomes containing at least 95% of the defined cgMLST targets were included. Isolates with less than 12 different alleles in the cgMLST target gene set were considered as highly related (and termed a cluster). Additionally, based on the assembled genomes, the conventional 7-loci MLST scheme was retrieved from the MLST database.^17^ Furthermore, acquired resistance genes on assembled genomes were identified by the ResFinder Bacterial Analysis Pipeline v. 2.1.^18^

The genetic environment of *bla*_VIM-2_ was annotated and curated manually and visualized using the SnapGene® software (Insightful Science, GSL Biotech, San Diego, CA, USA) based on the hybrid assemblies. Insertion sequence elements were investigated using the ISfinder database (http://www-is.biotoul.fr).^19^

### Infection prevention and control (IPC) management

A general rectal admission screening for MDR Gram-negative organisms was in place on all units. Additionally, weekly tracheal secretions were taken from intubated patients, and wound swabs from burn patients (surveillance cultures). Weekly rectal screenings were performed on ICU1 only. Standard and contact precautions were applied for every patient found colonized or infected with MRD/XDR *P. aeruginosa* (single room and use of gowns and gloves). Relevant clinical and epidemiological data was collected from patients’ clinical records or the attending physician. If the collection of the specimen occurred on or before the second day of admission, and there was no prior contact to the healthcare system within the previous 30 days, bacterial isolates were considered community-acquired. If prior contact with the healthcare system (other than our hospital) was observed within the previous 30 days and collection occurred on or before the second day of admission, isolates were considered healthcare-associated. If the collection of the specimen occurred after the second day, or if the patient stayed at our hospital within the last 30 days, bacterial isolates were defined as hospital-acquired. Transmission analysis was based on epidemiological data (direct room or ward contact, and/or documented care by the same staff) and genetic data. Proven transmission events were defined as isolation of genetically related isolates (cluster) in two or more patients who were hospitalized during overlapping periods on the same ward (at least 24 h, patient-to-patient transmission) or in the same room with a maximum time interval of 6 months (room-to-patient transmission).^12^ An interval of 6 months was chosen because transmission of *P. aeruginosa* from environmental sources can continue over longer periods and can be sporadic.^8,12^ Hospital-acquired infections were classified according to the CDC/NHSN definitions.^20^ Patients without related signs of infection were considered to be colonized.

### Ethics approval and consent to participate

The study was approved by the Ethics Committee of the Faculty of Health of the Witten/Herdecke University (study number S-33/2021).

## Results

### Isolate and patient characteristics

Seventy-nine first MDR/XDR *P. aeruginosa* clinical and screening isolates were detected from the designated wards during the time period and 55 non-duplicate isolates were available for further analysis.

Susceptibility testing by broth microdilution showed that all bacterial isolates displayed an MDR or XDR phenotype as defined by the inclusion criteria; no isolate was pandrug resistant. One isolate was not cultivable for broth microdilution. The remaining 54 isolates displayed a susceptibility rate of 50%, 61.1% and 100% for tobramycin, amikacin and colistin, respectively. In all MBL-producing isolates (*n *= 20) the susceptibility rate for aztreonam was 85% and was much lower in non-MBL-producing isolates (*n *= 34), being 26.4%. All MBL-producing isolates were resistant to ceftolozane/tazobactam and ceftazidime/avibactam, whereas in all non-MBL-producing isolates, susceptibility rates to ceftolozane/tazobactam and ceftazidime/avibactam were both 72.7%. MICs are shown in Table S1 (available as Supplementary data at *JAC-AMR* Online).

Sixteen of these isolates were previously analysed by conventional genotyping methods.^12^ Sequence analysis confirmed 20 carbapenemase-producing *P. aeruginosa* isolates as follows: *bla*_VIM-1_ (*n *= 1), *bla*_VIM-2_ (*n *= 17), *bla*_VIM-4_ (*n *= 1) and *bla*_NDM-1_/*bla*_GES-5_ (*n *= 1). Other relevant acquired resistance genes are shown in Table S1.

As all patients were from critical care units, devices, surgical and nonsurgical interventions and antibiotic therapy were common (Table 1). The mode of acquisition was mostly either hospital-acquired or healthcare-associated, and only one isolate was considered as community-acquired.

**Table 1.** Characteristics of 55 patients with the analysed MDR/XDR *P. aeruginosa*

Patient characteristics (*n *= 55)	Value
Age, years, median (Q1–Q3)	59 (46–70)
Sex, male	40 (72.7)
Medical departments	
surgery	32 (58.2)
internal medicine	23 (41.8)
Mode of acquisition	
hospital-acquired	42 (76.4)
healthcare-associated	12 (21.8)
community-acquired	1 (1.8)
Day of acquisition during hospital stay (hospital-acquired only; *n *= 42), median (IQR)	29 (30.25)
Hospital-acquired infection (CDC/NHSN)	
pneumonia	11 (20)
surgical site	5 (9.1)
urinary tract	2 (3.6)
skin	2 (3.6)
CLABSI	1 (1.8)
Antipseudomonal antibiotic treatment^a^	38 (69.1)
Surgery^a^	40 (72.7)
Nonsurgical intervention^a^	51 (92.7)
Dialysis^a^	18 (32.7)
Mechanical ventilation^a^	48 (87.3)
Central line^a^	47 (85.5)
Urinary catheter^a^	50 (90.9)

Values are shown as *n* (%) unless specified otherwise.

Q1, lower quartile; Q3, upper quartile; CLABSI, central line-associated bloodstream infection.

a Within a maximal interval of 7 days before first isolation of MDR/XDR *P. aeruginosa*.

### Genotyping and transmission analysis

**Figure 1.**
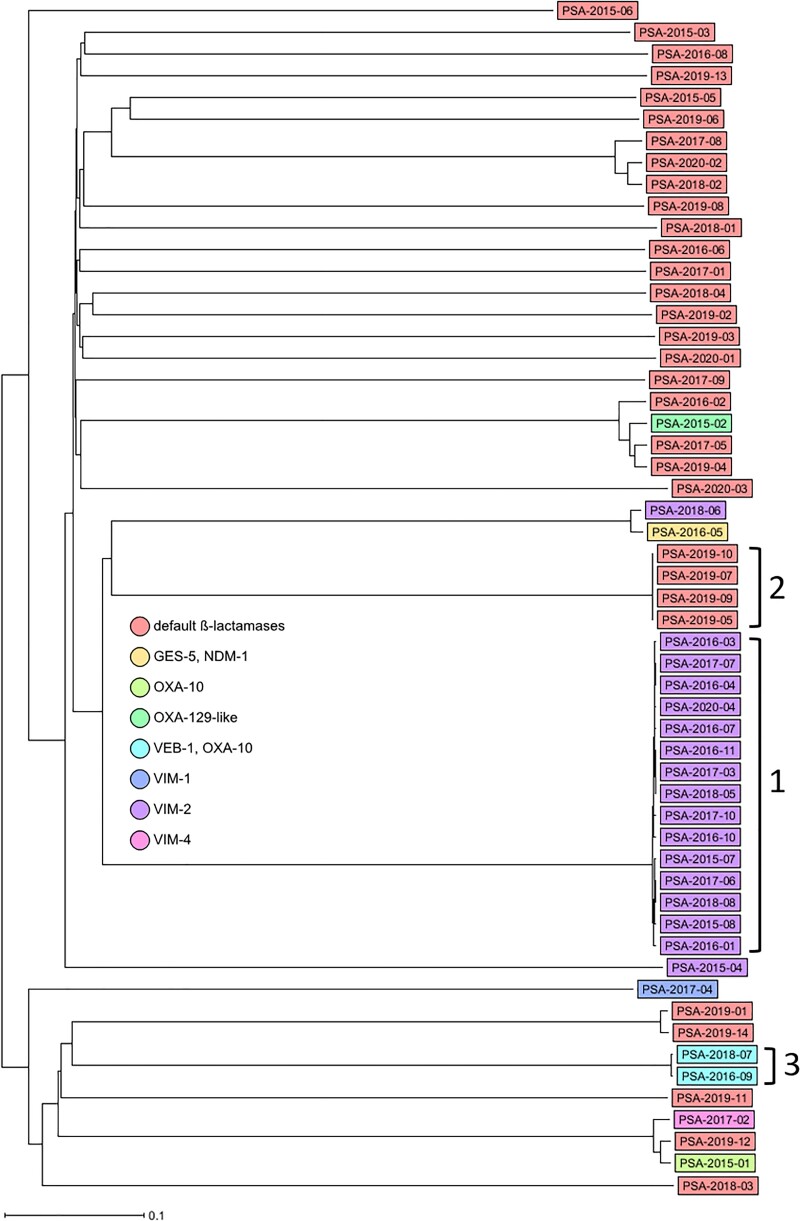
Ridom SeqSphere+ neighbour joining tree for 55 samples based on 3867 columns, pairwise ignoring missing values, percentage columns difference. Isolates are coloured based on their carbapenem-resistance mechanism. ‘Default β-lactamases’ is defined as those where only the *bla*_OXA-50-like_ and *bla*_PAO-like_ genes were detected. Clonal Clusters 1 to 3 are indicated by brackets and numbers.

**Figure 2.**
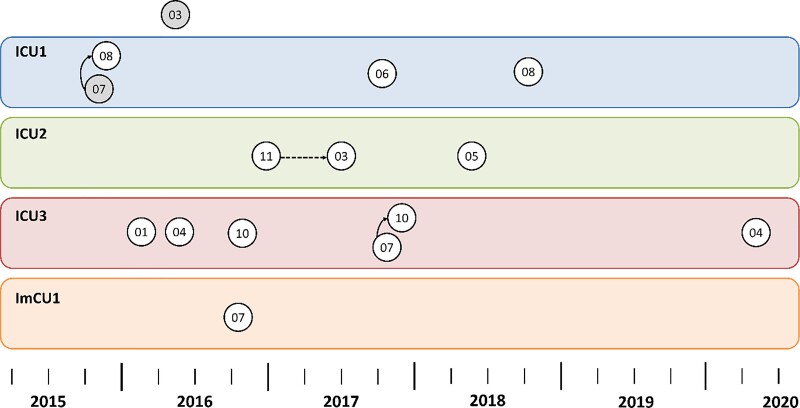
Epidemiological timeline and transmission route of Cluster 1 VIM-2-producing *P. aeruginosa*. Each circle represents one patient at time of first isolation. An arrow indicates genetically and epidemiologically confirmed transmission events (dashed line, room-to-patient; continuous line, patient-to-patient). Wards of transmission are indicated in different colours. Circle colour indicates mode of transmission (grey, healthcare-associated, white, hospital-acquired). Isolate numbers can be inferred by combining year and number in the node. Isolate no. PSA-2016-03 was isolated on another ward than the four critical care units.

Carbapenemase-producing *P. aeruginosa* isolates (*n *= 20) were assigned to five STs, predominantly ST111, but also ST273, ST654, ST235 and ST3618, the latter being a newly assigned ST. A high diversity was detected within the remaining 35 non-carbapenemase-producing *P. aeruginosa* isolates, which comprised 24 different STs. cgMLST revealed three clusters that were represented by different STs: Cluster 1 (*n *= 15, ST111), Cluster 2 (*n *= 4, ST970) and Cluster 3 (*n *= 2, ST357). Within Clusters 1, 2 and 3, a maximum difference of 21, 1 and 5 alleles, respectively, was observed. Carbapenemase production in *P. aeruginosa* was significantly associated with belonging to a clonal cluster (*P *< 0.001, Fisher’s exact test). Table 2 summarizes isolate characteristics, genotyping results and presence of ESBL/carbapenemase genes, and Figure 1 shows the relatedness of the isolates. All Cluster 1 isolates carried the *bla*_VIM-2_ gene and the *aacA29*-like gene. Moreover, all but two isolates from Cluster 1 were hospital-acquired; the other two were healthcare-associated and had contact with two different institutions. Cluster 1 isolates were detected sporadically on all four wards and during the whole study period from 2015 to 2020. Using epidemiological data, we were only able to confirm one room-to-patient and two patient-to-patient transmissions (on three different ICUs and during three different time periods; Figure 2).

**Table 2.** Characteristics of MDR/XDR *P. aeruginosa* ranked by cgMLST cluster type and date of isolation

Isolate no.	Date (month-year)	Specimen type	Epidemiological link to ward	ST	cgMLST cluster	Acquired β-lactamase genes
PSA-2015-07	Dec-15	wound	ICU1	ST111	1	*bla*_VIM-2_
PSA-2015-08	Dec-15	respiratory tract	ICU1			
PSA-2016-01	Feb-16	urine	ICU3			
PSA-2016-03	Apr-16	respiratory tract	other			
PSA-2016-04	Apr-16	respiratory tract	ICU3			
PSA-2016-07	Oct-16	respiratory tract	ImCU1			
PSA-2016-10	Dec-16	respiratory tract	ICU3			
PSA-2016-11	Dec-16	unknown	ICU2			
PSA-2017-03	Jul-17	screening (rectal)	ICU2			
PSA-2017-06	Oct-17	wound	ICU1			
PSA-2017-07	Oct-17	screening (nose/throat)	ICU3			
PSA-2017-10	Dec-17	respiratory tract	ICU3			
PSA-2018-05	Jun-18	urine	ICU2			
PSA-2018-08	Nov-18	wound	ICU1			
PSA-2020-04	Apr-20	screening (rectal)	ICU3			
PSA-2019-05	Aug-19	respiratory tract	ICU1	ST970	2	—
PSA-2019-07	Sep-19	screening (nose/throat)	ICU1			
PSA-2019-09	Sep-19	wound	ICU1			
PSA-2019-10	Sep-19	screening (rectal)	ICU1			
PSA-2016-09	Nov-16	respiratory tract	ICU2	ST357	3	*bla*_OXA-10*-*like_, *bla*_VEB-1_
PSA-2018-07	Nov-18	respiratory tract	ICU3			
PSA-2015-01	Jan-15	screening (rectal)	ICU1, ICU3	ST235	singleton	*bla*_OXA-10_
PSA-2015-02	Feb-15	wound	ImCU1	ST395	singleton	*bla*_OXA-129*-*like_
PSA-2015-03	Apr-15	screening (nose/throat)	ICU1	ST1233	singleton	—
PSA-2015-04	Jun-15	screening (rectal)	ICU2	ST273	singleton	*bla*_VIM-2_, *bla*_ACT-5*-*like_
PSA-2015-05	Oct-15	respiratory tract	ICU2	ST980	singleton	—
PSA-2015-06	Oct-15	wound	ICU3	ST17	singleton	—
PSA-2016-02	Mar-16	respiratory tract	ImCU1	ST395	singleton	—
PSA-2016-05	Sep-16	urine	ICU3	ST654	singleton	*bla*_NDM-1_, *bla*_GES-5_
PSA-2016-06	Oct-16	respiratory tract	ImCU1	ST918	singleton	—
PSA-2016-08	Nov-16	wound	ICU3	ST1743	singleton	—
PSA-2017-01	Jan-17	respiratory tract	ICU2	ST1044	singleton	—
PSA-2017-02	Feb-17	screening (rectal)	other	ST235	singleton	*bla*_VIM-4_
PSA-2017-04	Aug-17	urine	ICU2, ImCU1	ST3618	singleton	*bla*_VIM-1_
PSA-2017-05	Oct-17	screening (nose/throat)	ICU1	ST395	singleton	—
PSA-2017-08	Oct-17	respiratory tract	ICU3	ST274	singleton	—
PSA-2017-09	Oct-17	wound	ICU1	ST2069	singleton	—
PSA-2018-01	Jan-18	wound	ICU1	ST2167	singleton	—
PSA-2018-02	Feb-18	respiratory tract	ICU1	ST274	singleton	—
PSA-2018-03	Mar-18	respiratory tract	ICU2	ST701	singleton	—
PSA-2018-04	May-18	wound	ICU1	ST291	singleton	—
PSA-2018-06	Nov-18	urine	ICU2, ICU3	ST654	singleton	*bla*_VIM-2_
PSA-2019-01	Feb-19	respiratory tract	ICU2, ImCU1	ST207	singleton	—
PSA-2019-02	Mar-19	screening (nose/throat)	ICU1, ICU3	ST3480	singleton	—
PSA-2019-03	Jul-19	screening (rectal)	ImCU1	ST1320	singleton	—
PSA-2019-04	Jul-19	respiratory tract	ImCU1	ST395	singleton	—
PSA-2019-06	Aug-19	screening (rectal)	ICU1	ST508	singleton	—
PSA-2019-08	Sep-19	respiratory tract	ICU2	ST2332	singleton	—
PSA-2019-11	Oct-19	respiratory tract	ICU2	ST309	singleton	—
PSA-2019-12	Oct-19	screening (rectal)	ImCU1	ST235	singleton	—
PSA-2019-13	Nov-19	screening (rectal)	ICU3	ST27	singleton	—
PSA-2019-14	Nov-19	screening (rectal)	ICU3	ST207	singleton	—
PSA-2020-01	Jan-20	respiratory tract	ICU2	ST399	singleton	—
PSA-2020-02	Feb-20	screening (nose/throat)	ICU1	ST274	singleton	—
PSA-2020-03	Apr-20	respiratory tract	ICU2, ImCU1	ST871	singleton	—

PSA, *P. aeruginosa*; ST, sequence type (conventional 7-loci MLST).

Clusters 2 and 3 represented non-carbapenemase-producing isolates. Cluster 2 isolates were obtained from four patients, all from the same ward, with three proven patient-to-patient transmissions. We were not able to identify the transmission route of the index patient. This single outbreak among burn patients was actively identified by the IPC team at the time of detection and immediately terminated. The Cluster 3 isolates both harboured the ESBL genes *bla*_VEB-1_ and *bla*_OXA-10_, and differed by five alleles suggesting a transmission event, however the two patients had no known epidemiological link.

Singletons were generally separated from all other isolates by over 2785 alleles. Exceptions to this were the two ST207 isolates, the two ST654 isolates, the three ST235 isolates, the three 274 isolates and the four ST395 isolates, which differed by 39, 59, 96, 151 and 169 alleles, respectively.

We compared our Cluster 1 *bla*_VIM-2_-harbouring ST111 isolates with those from previous studies in the UK and the Netherlands.^21,22^ Raw reads were downloaded from the European Nucleotide Archive with the study number ERP010395 and PRJEB39528, and assembled as described in the materials and methods. The resulting minimum spanning tree is shown in Figure S1. The isolates from Germany clustered closely with the isolates from the UK and the Netherlands, with 19–58 allelic differences.

### Genetic environment of the MBL *bla*_VIM-2_

**Figure 3.**
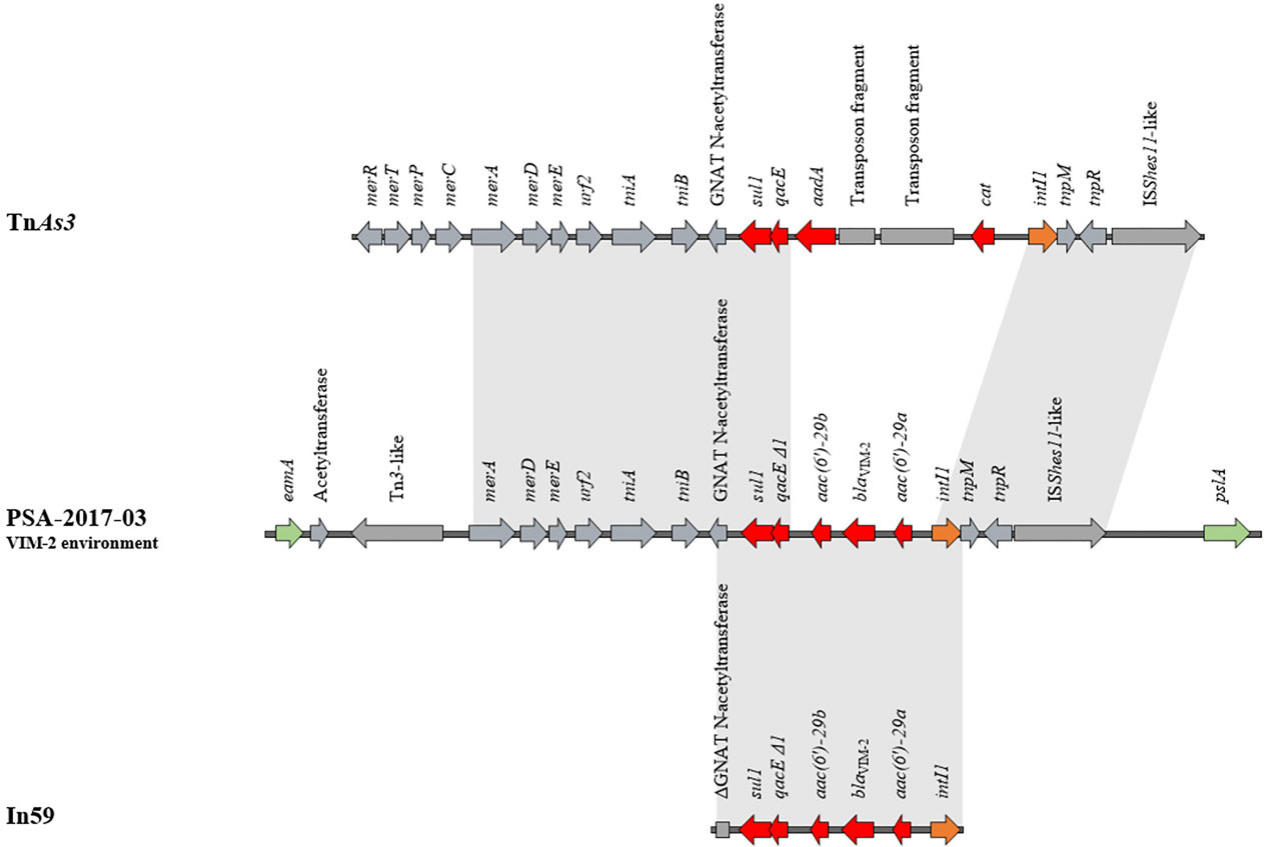
Schematic diagram of the genetic environment of *bla*_VIM-2_ in the isolate PSA-2017-03 compared with the transposon Tn*As3* (accession number CP000645) and the integron In59 (accession number AF263519.1). Arrows indicate the deduced open reading frames (ORFs) and their orientations. Homologous regions are shaded in grey. Antimicrobial resistance genes are shown in red and the *intI1* gene is shaded orange. The ORFs EamA family transporter (putative membrane protein) and PslA (biofilm formation protein) coloured in green represent chromosomal markers. The remaining genes are shown in grey.

Hybrid genome assemblies (short and long reads) of three ST111 VIM-2-producing isolates from the cgMLST Cluster 1 revealed that the genetic environment of the *bla*_VIM-2_ gene was identical in the three isolates: a class 1 integron located in the chromosome. The *bla*_VIM-2_ gene was flanked by aminoglycoside resistance genes *aacA(6′)-29a* and *aacA(6′)-29b*, differing in four amino acid substitutions, as well as the integrase gene *intI1*. Further upstream the antiseptic resistance cassette *qacE* was detected, followed by *sul1* conferring resistance to sulphonamides. The genetic environment of the MBL was highly similar (blastn coverage 100% and identity 99.5%, accession number AF263519.1) to the integron In59.^23^ Finally, the integron was inserted into a transposon Tn*As3*-like structure (blastn coverage 72% and identity 99%, accession number CP000645) (Figure 3). Tn*As3* belongs to the Tn*3* family and the Tn*21* subgroup.^24^

## Discussion

*P. aeruginosa* is an important hospital-acquired pathogen causing infections and outbreaks in ICUs.^1^ Over the last few years we have seen a dramatic increase of MDR isolates worldwide.^2,3,25^ In this study, from a collection of MDR/XDR *P. aeruginosa* isolates from four critical care units of a tertiary care centre transmission was nearly exclusively observed among ST111 VIM-2-producing isolates over a period of 5 years. Another short-term outbreak caused by ST970 non-carbapenemase-producing *P. aeruginosa* was observed in vulnerable patients in the burn unit. We observed a higher diversity in non-carbapenemase-producing isolates, most of the isolates being genetically unrelated to each other. Overall, the study reveals a remarkable clonal diversity, with most isolates represented by sporadic single genotypes, and a few epidemic strains.

Studies comparing clonal diversity among susceptible and MDR/XDR resistant isolates have shown a lower diversity among MDR and especially XDR strains and that XDR/MDR *P. aeruginosa* infections are disproportionally caused by a small subset of globally distributed ‘high-risk clones’, linked to mutational resistance determinants but also transferable resistance genes.^4,26–28^ Traditionally, these clones are classified according to the MLST scheme developed by Curran *et al.*^29^ over 15 years ago. Four out of the worldwide top 10 *P. aeruginosa* high-risk clones (ST235, ST111, ST357 and ST654) were found in this study.^4^

In our study the appearance of high-risk clones was also linked to carbapenemase production, mostly ST111 having acquired VIM-2, a combination that has also been described previously.^4,30^ Other surveillance studies from the UK and the Netherlands reported that ST111 *P. aeruginosa* was linked mostly to VIM-2 production.^21,22^ Epidemiological data and high resolution genotyping by WGS showed evidence for spread within and between hospitals in different regions of the UK.^21^ It is important to note that in our study most isolates were hospital-acquired and a limited number of isolates were also present on admission and healthcare-associated. As there is no continuous molecular surveillance on a regional or national level in Germany, we are unable to determine the extent to which ST111 carrying carbapenemases has spread throughout the region. However, VIM-2 is the leading carbapenemase in *P. aeruginosa* based on data from the German national reference centre.^31^ Moreover, previous studies have shown ST111 VIM-2-producing *P. aeruginosa* in Hamburg in 2001,^32^ and Wendel *et al.*^8^ reported the spread of ST111 GIM-1-producing *P. aeruginosa* in a hospital located close to Cologne. Furthermore, we also demonstrated that the ST111 isolates from the UK and the Netherlands clustered closely, although not overlapping, with those from the current study, highlighting that these high-risk clones are not confined to small geographical regions but have in fact spread to other countries. The genetic environment of the *bla*_VIM-2_ gene of the ST111 clone was highly similar to the integron In59 described in a VIM-2-positive *P. aeruginosa* isolate recovered in 1997 in France and since then in different European countries, and mostly in ST111 isolates.^23,33–35^ As high-risk clones tend to harbour several resistance traits they are also linked to aminoglycoside resistance, with the *aacA29a* gene being the most common determinant in ST111, also confirmed in our study.^30^

Microbiological and infection control monitoring of carbapenem resistance in *P. aeruginosa* is of utmost importance with regard to the clonal structure and mobile genetic elements such as carbapenemases. Both SNP-based and cgMLST-based typing have been successfully applied in various studies.^9,10,36–39^ Recently, several validated cgMLST schemes were published enabling a standardized approach and a consistent nomenclature.^16,40,41^ The cgMLST scheme proposed by Tönnies *et al*.^16^ (3867 targets) used in this study was comparable to an ad-hoc cgMLST scheme previously established by one of the authors (4547 targets).^39^

Depending on the mode of transmission, different infection and control approaches are needed. Individual nosocomial acquisition of *P. aeruginosa* is either endo- or exogenous and can subsequently lead to transmission chains. Sporadic or low-frequency transmissions from the environment (mostly moist sites) to the patient are difficult to trace back epidemiologically as shown in several studies.^8,10,11^ However, we were unable to confirm many transmission events within the hospital despite sporadic appearance of the clone. A hidden environmental reservoir (especially sinks) or complex epidemiological links might be an explanation. There is growing evidence supporting a water-free patient environment and removing sinks from the patient’s room to eliminate patient-side biofilm reservoirs.^9,42,43^ This is possible on the ICU where the patient generally does not need a bathroom. Nevertheless, we did not find a pattern of room transmissions as patients from the biggest cluster were found on all four wards.

There are a few limitations in this study. Unfortunately, we were not able to provide full prevalence data, as only two-thirds of the non-duplicate isolates detected during this period were available for further study. Moreover, transmissions by unidentified colonized patients might have been overlooked as there was no periodic rectal screening in place on all units. However, we are confident that the study gives a good overview of the epidemiological pattern. Secondly, the inclusion criteria were probably not sensitive enough to detect carbapenemases, as we chose to include isolates based on the German classification guideline for Gram-negative MDR organisms (Gram-negative MDR organisms with resistance to four out of four major antibiotic classes, 4MRGN).^44^ However, carbapenemase production is often linked to MDR/XDR phenotypes.^45^

Thirdly, the mutational resistome was not analysed and we only performed analysis of acquired/intrinsic resistance genes *in silico* using a web-based tool. This is more complex and not completely validated yet and out of the scope of our investigation, which is basically epidemiological.^46^ Fourthly, we did not conduct environmental sampling in this study to detect an inanimate reservoir; however we want to point out that on two out of four of the ICUs, sinks in most of the patient rooms were removed during the study period.

In conclusion, to ensure surveillance of *P. aeruginosa* high-risk clones and carbapenemase genes, it is necessary to implement diagnostic tools at local level for their early detection and the combination with epidemiological data, in order to guide IPC strategies. This is especially the case among carbapenemase-producing high-risk clones that were associated with ongoing acquisitions. Therefore, the timely detection of carbapenemases can potentially lead to strategies to halt transmission.

## Supplementary Material

dlac057_Supplementary_Data

## Supplementary data

Table S1 and Figure S1 are available as Supplementary data at *JAC-AMR* Online.
