# Supplementary figures and images for: Genomic-based transmission analysis of carbapenem-resistant Pseudomonas aeruginosa at a tertiary care centre in Cologne (Germany) from 2015 to 2020
Source: JAC Antimicrob Resist. 2022 May 20;4(3):dlac057. doi: 10.1093/jacamr/dlac057 (PMC9122648; doi:10.1093/jacamr/dlac057)

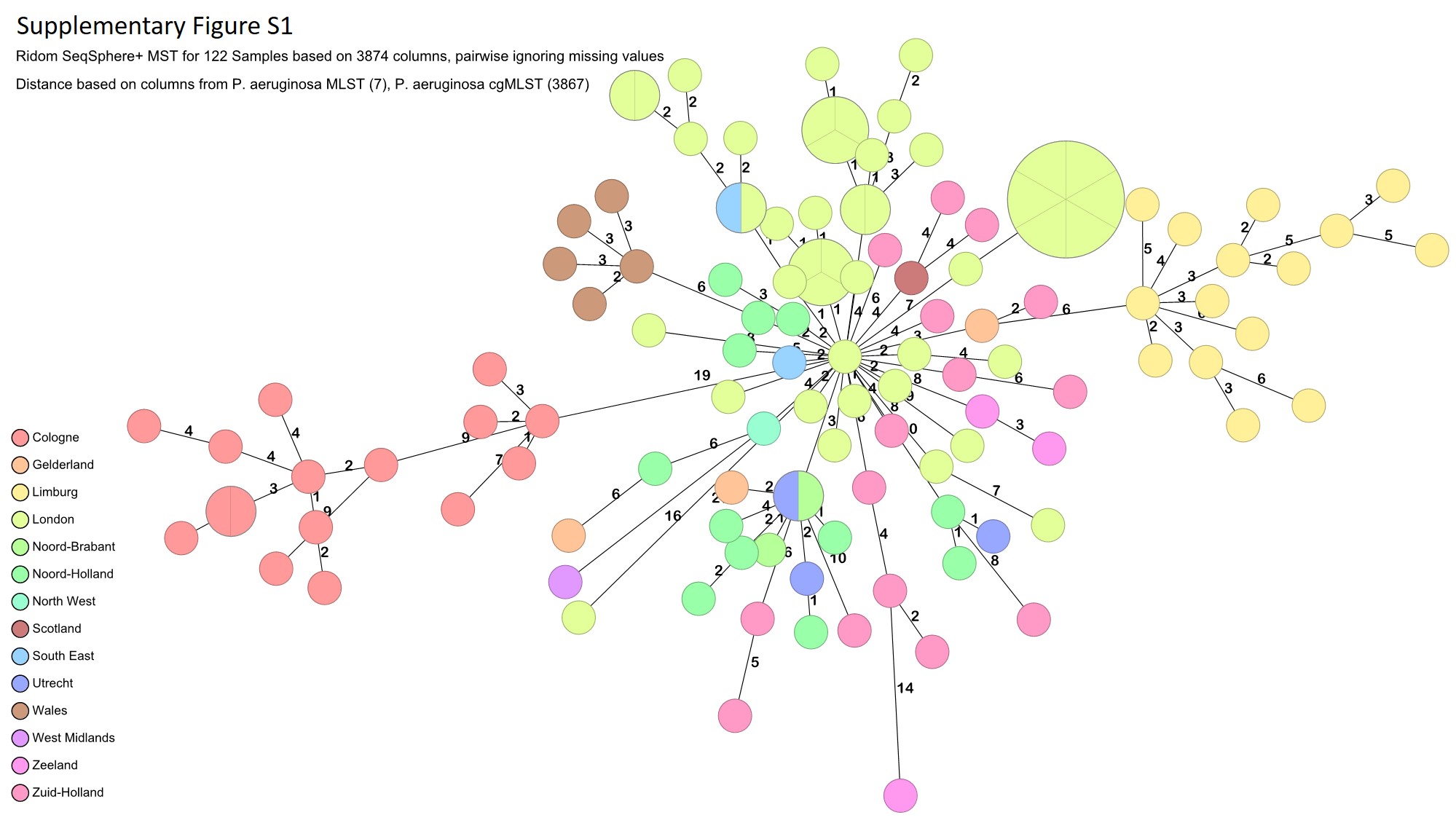

Supplement: dlac057_Supplementary_Data [file dlac057_supplementary_data.zip › Figure_S1.jpg]
